# Graphene Oxide Membrane Immobilized Aptamer as a Highly Selective Hormone Removal

**DOI:** 10.3390/membranes10090229

**Published:** 2020-09-12

**Authors:** Siham Chergui, Khaled Rhili, Sujittra Poorahong, Mohamed Siaj

**Affiliations:** Department of Chemistry, University of Quebec at Montreal, Montreal, QC H3C3P8, Canada; chergui.siham@courrier.uqam.ca (S.C.); rhili.khaled@courrier.uqam.ca (K.R.); sujittrapoorahong@gmail.com (S.P.)

**Keywords:** graphene oxide, polyethyleneimine, aptamer, graphene membrane, dexamethasone

## Abstract

Three-dimensional (3D) reduced graphene oxide (rGO) modified by polyethyleneimine (PEI) was prepared and functionalized by fluorophore-labeled dexamethasone-aptamer (Flu-DEX-apt) via π–π stacking interaction. The rGO/PEI/Flu-DEX-apt was used as a selective membrane for dexamethasone hormone removal from water. The prepared rGO/PEI/Flu-DEX-apt membranes were stable, insoluble, and easily removable from liquid media. The membrane was characterized by Raman spectroscopy, scanning electron spectroscopy, and FTIR spectroscopy. The rGO/PEI/Flu-DEX-apt membrane showed high sensitivity and specificity toward the dexamethasone hormone in the presence of other steroid hormone analogs, such as progesterone, estrone, estradiol, and 19-norethindrone. The fluorescence and UV–visible spectroscopy were used to confirm the membranes performance and the quantification of hormones removal. The resulting data clearly show that the graphene oxide concentration influence the aptamers and analytes interaction (π–π stacking interaction). It was found that by varying the graphene oxide concentration yields to different porosities of rGO/PEI/Flu-DEX-apt membranes affects the adsorption recovery rate, as well as the specificity and selectivity toward the dexamethasone hormone.

## 1. Introduction

Graphene possesses a high surface area, thermal conductivity, outstanding mechanical properties, and electron mobility higher than conductor material [1,2]. Since its discovery in 2004 [3], graphene has played an important role in different fields as much as a carbon nanomaterial. Graphene and graphene oxide (GO) can be synthesized through different methods like chemical oxidation route and chemical vapor [4] deposition (CVD) [5,6]. The successive oxidation of graphite leads to graphite oxide [5], with a structure containing oxygen functional groups such as carboxylic acid at its edges while the basal plane bears epoxy and hydroxyl groups [7]. These functional groups made the graphene oxide have a hydrophilic behavior and can also serve as a base to form covalent bonds with other functional groups, for example by esterification (COO-R) or amidization (CONH-R) [8]. Thus, GO has better solubility in various solvents [9,10]. In addition, GO exhibits fluorescence quenching behavior towards biomolecules which could be used to understand their dynamics change during the interaction between the biomacromolecular system and GO surfaces. Fluorescence quenching effects have been used successfully in biology and medicine applications such as biological imaging and to monitor an in situ transport medium for drug administration [11,12]. The non-covalent interaction such as hydrogen bonding, Van der Waals forces, cation–π linkage, and π–π stacking occurring between the GO and biomolecules could be used as a selective membrane in separation techniques [4,13]. Selectivity arises as an issue since many molecules can undergo these interactions. Successful theoretical approaches showing the modification of the graphene surface and the interaction between the graphene-derivative-like systems made available through density-functional ab initio molecular dynamics [14] density-functional theory (DFT) calculations [15]. The systematic study reveals the interplay between the defects and disruptions resulting from the incorporation of adsorbed atoms in graphene-like networks and how their interaction defines the structural features of these films. The zwitterion behavior of GO leads to strong interactions between biomolecules (proteins) and biopolymers (DNA, ssDNA) [16,17]. Functional groups of graphene oxide are naturally interactive, and may covalently bind to small molecules or polymers leading to nanocomposite formation such as polymer fillers (e.g., polyvinyl alcohol (PVA), poly(methyl methacrylate) (PMMA) or polyethyleneimine (PEI) [18,19]. For this to build a selective membrane, a functionalization of the GO/PEI surface with aptamers seems a plausible route to reach high adsorption and selective separation for desired analytes. Aptamers adsorb highly to the graphene oxide surfaces through the π–π interaction yielding to direct changes on the fluorescence quenching behavior. Thus, successful interaction between rGO/PEI/fluorophore-labeled dexamethasone-aptamer (Flu-DEX-apt) and the targeted analyte could be monitored by the fluorescence signal. 

Herein, we reported the synthesis of a stable, biocompatible and robust 3D porous rGO/PEI/Flu-DEX-apt membrane [20,21]. The prepared rGO/PEI/Flu-DEX-apt foam exhibited excellent stability in the solution. The PEI plays a key role in obtaining a 3D structured porous foam that can be reused easily [19]. Modified rGO/PEI foams with aptamers foresee possible applications as a selective membrane for water treatment. Different graphene oxide concentrations have been used for membrane preparation. We found that the amount of the GO directly affects the pore size distribution of the resulting foam, leading to a better ssDNA penetration and loading and therefore, an increased selectivity. The developed fluorophore-labeled dexamethasone-aptamer (Flu-DEX-apt) was used as a model for specific hormones’ (endocrine disruptors) removal from water as emerging contaminants. The rGO/PEI and Flu-aptamers interactions were confirmed by fluorescence spectroscopy. The dexamethasone hormone removal increased systematically by increasing the GO concentrations on the fabricated membranes. A labeled dexamethasone aptamer membrane allowed a high specificity and selectivity. For cross specificity and selectivity testing, the analogs of hormones like progesterone, estrone, 19-norethindrone and estradiol were used under the same condition dexamethasone hormone removal. In this work, a combination of GO, PEI, and Flu-DEX-apt leads to a stable membrane for dexamethasone hormone filtration.

## 2. Experimental Section 

### 2.1. ssDNA Aptamer 

The systematic evolution of ligands by exponential enrichment (SELEX) method used to produce a specific single-stranded DNA (ssDNA), dexamethasone-aptamer. The fluorophore-labeled dexamethasone-aptamer (Flu-DEX-apt) is composed of 59 nucleotides. Dexamethasone-aptamer was labeled with fluorescein isothiocyanate (FITC) fluorophore: Fluor: 5’-/5FluorT/AC-ACG-ACG-AGG-GAC-GAG GAG-TAC-TTG-CCA-ACG-ATA-ACG-TCG-TTG-GAT-CTG-TCT-GTG-CCC-3’ [21]. Different hormones i.e., 19-norethindrone, estrone, dexamethasone, progesterone, and estradiol were purchased from Sigma-Aldrich (Oakville, ON, Canada).

### 2.2. Graphene Oxide Synthesis

The GO synthesis was made through the modified Hummers method [5]. First, concentrated H_2_SO_4_ (360 mL) and 85% H_3_PO_4_ (40 mL) were incorporated with graphite flakes (3 g). Afterward, KMNO_4_ (18 g) was mixed for 15 min. The obtained product was submitted to vigorous stirring at 55 °C for 4 h. In order to facilitate the chemical exfoliation of the graphite oxide, the mixture was put under sonication for 20 min at room temperature (four times). The addition of H_2_O_2_ (30 %) in the solution in the presence of an ice bath allowed its neutralization, hence the color changed to bright yellow. The elimination of metallic impurities from the obtained solution was carried out under stirring and by adding the successive doses (twice) of water (1 L), 100 mL of 37% HCl solution, and twice with ethanol (2 L). After, the washing step solution was obtained as an orange-colored hydrogel. The separation of the GO sheets was carried out under stirring by the addition of ethanol (1 L), and then filtrated. the filtrated products were dried at 30 °C for 48 h to yield GO powder. Finally, graphene oxide was diluted in deionized water (4 g/L) under stirring before being preserved at room temperature.

### 2.3. Functionalized rGO@polyethyleneimine Preparation

The functionalization and the synthesis of a 3D membrane were carried out by the incorporation of the GO in the branched PEI [22]. Briefly, 10 mL solution of graphene oxide (4 g/L) was conjugated with 200 mg PEI in a hot oil bath (90 °C). The reaction was kept for 12 h and then it was placed at −80 °C for 4 h followed by a freeze-drying process for 72 to 86 h. Finally, the 3D membrane rGO/PEI was stored at 100 °C for 48 h. As the adsorption of biomolecules on GO is related to the membrane porosity and structure, different membranes were prepared based on different GO concentrations (C) of C_1_ = 0.500, C_2_ = 0.858, C_3_ = 1.358 and C_4_ = 3.716 g/L.

### 2.4. Apparatus and Characterizations

Scanning electron microscopy (SEM) (JEOL-JSM7600F, Peabody, MA, USA) was used to determine the morphology and the porosity of the rGO/PEI foam. Raman spectroscopy with alpha300R Confocal Raman Microscope with WITec UHTS 300 spectrometer (Ulm, Germany) with a 532 nm laser shows D and G bands characteristic of the graphene oxide. InfraRed spectroscopy (Nicolet 6700 FTIR, Madison, WI, USA) was used to determine rGO-PEI linking and the oxygen moieties presence. UV–vis Spectrophotometer (LAMBDA 750 UV/Vis/NIR Spectrophotometer (Perkin Elmer, Waltham, MA, USA), and fluorescence spectrometry (Perkin Elmer LS45 Spectrophotometer, Waltham, MA, USA) are used to monitor the adsorption behavior.

### 2.5. Developed Aptamer Specificity and Selectively Measurement

Aptamers’ specificity and selectivity were done using different steroids’ hormonal analogs such as progesterone, estrone, 19-norethindrone and estradiol. The concentration at 15 ppm of each analog was used for the study. The incubation was carried out for 1 h with rGO/PEI membrane bound aptamers.

### 2.6. Adsorption Efficiency

The incubation of the rGO/PEI membrane (obtained from different concentrations of GO) with 10 nM of Flu-DEX-apt in 1 mL binding buffer, pH 7.4 for 2 h at room temperature, under end-over-end rotation has been accomplished to prepare the rGO/PEI membrane/aptamer complexes. Then, a centrifugation step of the mixture was made to discard the non-binding aptamer and to obtain the rGO/PEI/Flu-DEX-apt membrane. The dexamethasone-aptamer, which bind graphene oxide foams through π–π stacking interaction, was mixed with dexamethasone analyte and incubated for 1 h. Meanwhile, free GO foam (without Flu-DEX-apt) was mixed with dexamethasone analyte as a control experiment. The resulting solution was examined by fluorescence and UV–visible measurements excited with a wavelength of 280 nm to study the adsorption efficiency and recovery rate.

## 3. Results and Discussion

### 3.1. Fluorescence Intensity Dependency on Graphene Oxide Concentration

**Figure 1:**
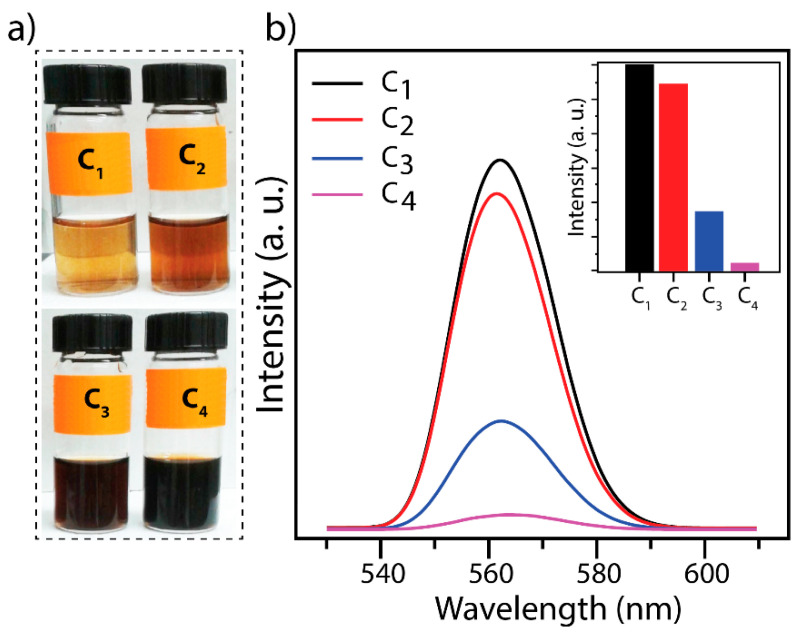
Fluorescence intensity dependence on graphene oxide concentration: (**a**) photographs of graphene oxide solutions with different concentrations (C), i.e., C_1_ = 0.500 g/L, C_2_ = 0.858 g/L, C_3_ = 1.358 g/L and C_4_ = 3.716 g/L, the color changes from nearly clear (low concentration) to dark (high concentration); (**b**) 280 nm was used as wavelength excitation to generate the fluorescence emission spectrum of different graphene oxide (GO) concentrations; the emission maximum is seen at 565 nm (the insert fluorescence intensity vs. the graphene oxide concentration).

Graphene oxide, given its peculiar optical properties, is an effective fluorescence quencher of labeled biomolecules [23,24], quantum dots, and organic dye molecules [25]. It was found that graphene oxide concentration directly affects fluorescence emission intensity. For this purpose, 10 nM ssDNA was conjugated with several concentrations (C) of graphene oxide, i.e., C_1_ = 0.500, C_2_ = 0.858, C_3_ = 1.358, and C_4_ = 3.716 g/L for studying the quenching effect of GO (Figure 1a). Figure 1b shows the fluorescence emission spectrum of GO at the concentrations of (0.500, 0.858, 1.358 and 3.716 g/L). The excitation wavelength was set at 280 nm. The fluorescence emission wavelength maximum was found at 565 nm as can be seen in Figure 1b. The fluorescence intensity was decreased with the increasing GO concentration. The variation of the fluorescence signal is influenced by the recombination of electrons and holes confined in sp^2^ carbon groups incorporated into the sp^3^ carbon structure [26]. The electrons and holes recombination act as chromophores or luminescence centers [27]. Indeed, the sp^3^ sites (defect) due to graphite oxidation has a direct effect on the fluorescence signal. Thus, by increasing the GO concentration, the sp^3^ sites (defect and disorder) systematically increase and reducing the fluorescence signal due to quenching phenomena. Thus, tuning the amount of sp^2^ sites in carbon structure will allow controlling the bandgap and consequently the fluorescence emission [28]. In this sense, the GO reduction leads to a variation in the fluorescence signal intensity which is attributed to the variation of the sp^2^ population [29,30].

### 3.2. Characterization of rGO/PEI Membrane

**Figure 2:**
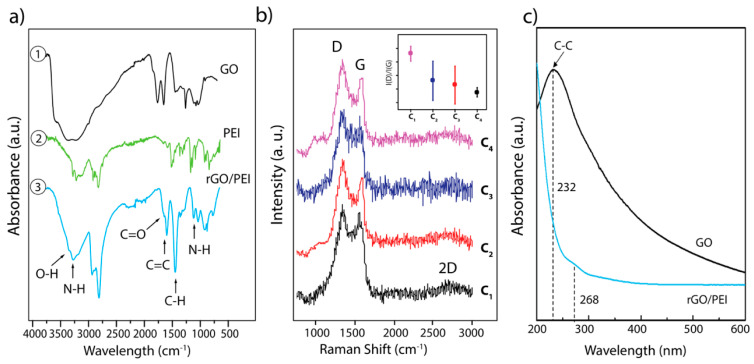
FTIR analysis spectra: (**a1**) spectrum of graphene oxide; (**a2**) spectrum of polyethyleneimine (PEI). (**a3**) spectrum of the combined product reduced graphene oxide (rGO)–PEI; (**b**) Raman spectra of the different rGO/PEI concentrations: i.e., C_1_ = 0.500 g/L, C_2_ = 0.858 g/L, C_3_ = 1.358 g/L and C_4_ = 3.716 g/L and the insert in Figure 2b shows the intensity ratio distribution plot of D and G peaks; and (**c**) the UV–visible spectra of GO and rGO/PEI.

The coupling between GO carboxyl groups (COOH–) and PEI amine groups (NH_2_–) was confirmed by FTIR spectroscopy, in which the characteristic amide (NH–CO) peak at 1108–1131 cm^−1^ (Figure 2(a3)) is very resolved. Figure 2(a1) showed the oxidation behavior of GO, which was confirmed by a broad characteristic stretching band of hydroxyl functional groups at 3354 cm^−1^ (C–OH) while an intense peak for the ketone group is located at 1725 cm^−1^ (C=O) and the alkene peak is located at 1606 cm^−1^ (C=C). Furthermore, the FTIR spectrum of PEI (Figure 2(a2)) showed the presence of peaks at 3293 cm^−1^ (–NH), 3000–2800 cm^−1^ (–C–H), and 1124 cm^−1^ (–C–N) stretching. The peak at 1629 cm^−1^ is attributed to the delocalized carbonyl group bond from the amide group in graphene oxide–polyethyleneimine composite and the vanishing of the C=O graphene oxide bands which corroborated the amide linkages formation (Figure 2(a3)). Moreover, the FTIR data exhibit a significant decrease in the intensity of the formed functional groups depending on the GO concentration. The C–N bond, which is generated between the PEI and the graphene oxide, is visible at 1124 cm^−1^ while the amine (NH–) band appears at 3293 cm^−1^ (Figure 2(a3)). This confirmed that the PEI is covalently linked to the GO carboxylic groups resulting in a 3D stable porous graphitic structure (see Figure 2(a3)).

**Figure 3:**
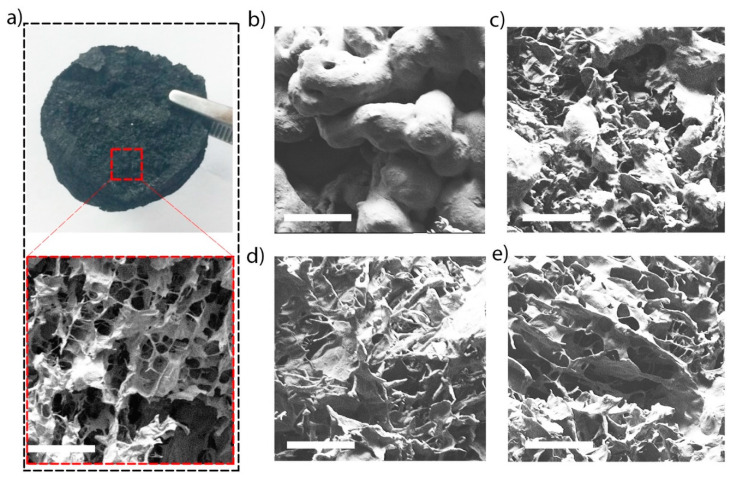
(**a**) Optical images of the reduced graphene oxide membrane and high magnification SEM image showing the 3D porous architecture membrane. The SEM images of 3D membrane graphene oxide–PEI foam made from different concentrations (C) of GO; (**b**) C_1_ = 0.500; (**c**) C_2_ = 0.858; (**d**) C_3_ = 1.358; and (**e**) C_4_ = 3.716 g/L (200 mm scale bar).

Figure 2b shows the Raman spectra of the rGO foam at different concentrations of GO. It displays two characteristic peaks of the carbon environment. The D peak around 1341 cm^−1^ and the G peak at around 1550 cm^−1^ are assigned to the number of defects and sp^2^ carbon bond vibrations, respectively. The presence of a 2D band around 2761 cm^−1^ with lower intensity (see Figure 2b), indicating that GO prepared in solution tends to aggregate and form a bulk graphite-like material in the absence of water [31]. The ratio between the D peak and G peak intensities *(I*_(D)_/*I*_(G)_) could indicate the amount of the defect in the rGO material. Measurements for these four samples of rGO were collected from five different areas, to construct the *I*_(D)_/*I*_(G)_ ratio distribution plot (Figure 2b). This difference in the ratio is probably due to the different angles of rotation between the constituent layers [32]. For this purpose, we observed a difference in the amounts of defects characterized by different ratios of intensity *I_(D)_/I_(G)_* in each rGO sample (see insert Figure 2b). The concentration 4 (C_4_) has the lower *I_(D)_/I_(G)_* ratio *=* 0.83, followed by C_3_ with *I_(D)_/I_(G)_* = 0.84; C_2_ with *I_(D)_/I_(G)_* = 0.85 and C_1_ with *I_(D)_/I_(G)_* = 0.86. Indeed, the difference in the ratio indicates that C_1_ presents more defects. However, due to the abundant carboxylic, epoxy and hydroxyl groups clustered amongst the GO flakes, all samples demonstrated defects. Unpredictably, sample C_4_ appears to have less defects compared to other samples. Moreover, the peak position of the G band increases with a higher value of *I_(D)_/I_(G)_*. This may be explained that the defects are localized mainly along the edges in high GO concentration, which could be confirmed from SEM images (Figure 3).

Figure 2c shows the UV–vis absorption spectra of the GO and rGO/PEI solution. The spectrum of GO showed an intense peak for the aromatic bond (C–C) at 232 nm relating to the π–π* transition [33]. After the coupling between GO and PEI, the peak at 232 nm disappeared and another peak was generated at 268 nm, which confirmed the reduction of GO to rGO by the presence of PEI [34].

SEM images (Figure 3) show the surface morphology depicting the significant impact of graphene oxide concentration on the 3D foam porosity. Graphene oxide concentration was critical to determine the pores sizes and their distribution. The four resulting foams (Figure 3a) conserved similar structures, aside from differing in the pore diameters. As the concentration of graphene oxide increased, the diameter and formation of the porosity are increased. At lower concentrations of 0.500 g/L graphene oxide, a more compact structure was observed, as compared to the foam product of high graphene oxide concentration (Figure 3b). When the graphene oxide concentration is increased to (0.858–1.358 g/L), the porosity delimitations is gradually clarified: however, their walls were randomly distributed (Figure 3c,d). At the high concentrated solution of graphene oxide (3.716 g/L), the resulting membrane shows a larger pore size and a more ordered distribution of graphene walls (Figure 3e).

### 3.3. Mechanism of DNA Adsorption on Reduced Graphene Oxide Foam

#### 3.3.1. Binding Mechanism

**Figure 4:**
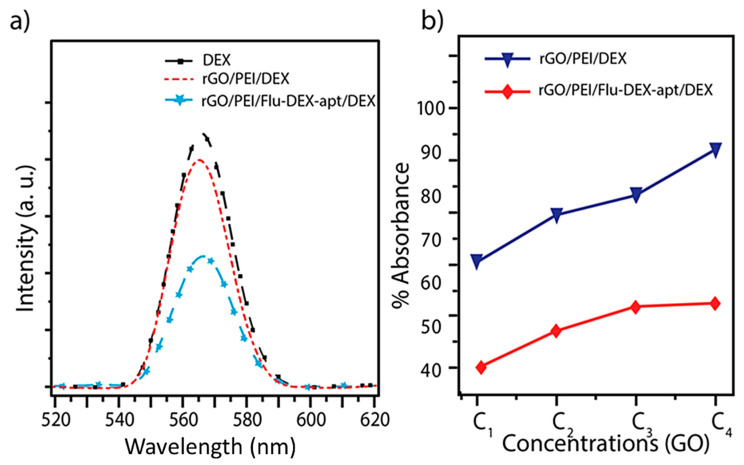
(**a**) Comparison of fluorescence intensity quenching efficiency between reduced graphene oxide–analyte/reduced graphene oxide–aptamer–analyte complex excited at λ_max_ = 280 nm; (**b**) percentage of the absorption intensity for rGO/PEI/fluorophore-labeled dexamethasone-aptamer (Flu-DEX-apt) and the rGO/PEI-Dex for different graphene oxide concentrations.

The adsorption of the analyte on the graphene oxide and binding affinities GO-ssDNA-hormone complex was analyzed by fluorescence spectroscopy. Since GO is capable of quenching adjacent emitting biomolecules, labeled ssDNA aptamers would find their fluorescence signal reduced once bound to the quencher [27,30,35]. The intensity of the fluorescence of DEX is decreased significantly after the aptamer was incubated for 2 h with rGO/PEI 3D membrane (Figure 4a). The rGO/PEI/Flu-DEX-apt foam was set to chelate dexamethasone as a targeted analyte. The fluorescence intensity decreased upon the addition of the Flu-DEX-apt (blue line) in contrast with the rGO/PEI/ DEX alone (red line). From this result, it clearly confirmed the adsorption of the aptamer on the graphene oxide foam and the binding between the aptamer and the analyte.

#### 3.3.2. Adsorption Efficiency

Aptamers are ssDNA that can be used as a recognition probe for specific water decontamination [36]. ssDNA affinity binding on rGO/PEI membrane was verified by the fluorescence quenching once the aptamer (Flu-DEX-apt) attached to the rGO/PEI foams. The fluorescence quenching was measured for each concentration of GO. The fluorescence spectrum of dexamethasone, rGO/PEI, dexamethasone-aptamer, and rGO/PEI/dexamethasone-aptamer revealed different behaviors following different interaction status between the graphene oxide, PEI and the Flu-DEX-apt (Figure 4). The intermolecular interactions and hydrogen bonding fortified these liaisons, and the fluorescence intensity quenching with graphene oxide was observed. For this purpose, the binding process between aptamer and dexamethasone analyte was carried out by several interactions such as Van deer Waals forces (weak intermolecular electrostatic interactions: dipole–dipole, ion–dipole and ion–ion), H-bonds formation, S-stacking and hydrophobic effect. These interactions between the aptamer and the analyte obey to the chemical equilibrium which is defined by a dissociation constant (Kd) [37].

With the incubation of the rGO/PET/Flu-DEX-apt 3D membrane with dexamethasone analyte, the fluorescence intensity signal increased significantly. The observed changes in the fluorescence signal after adding the ssDNA could be explained by the conformation changes of the Flu-DEX-apt, directly affecting the charges transfer from or/to the graphene surface. Thus, the affinity between the aptamer and the analyte (dexamethasone) could be confirmed.

In fact, the high specificity of aptamers to bind a target as biorecognition elements, confer their use in several applications such as sensors [38] and contamination removal membranes. In parallel, graphene-based membranes exhibit excellent molecular separation properties for purifying water [28]. Macroporous 3D graphene membranes are well suited for fast water purification and efficient desalination [39]. The percentage of absorption is shown in Figure 4b. As can be seen, the absorption efficiency increased by about ~30% by using the rGO/PEI/Flu-DEX-apt as a membrane compared to without DEX-apt at all concentrations of GO.

**Figure 5:**
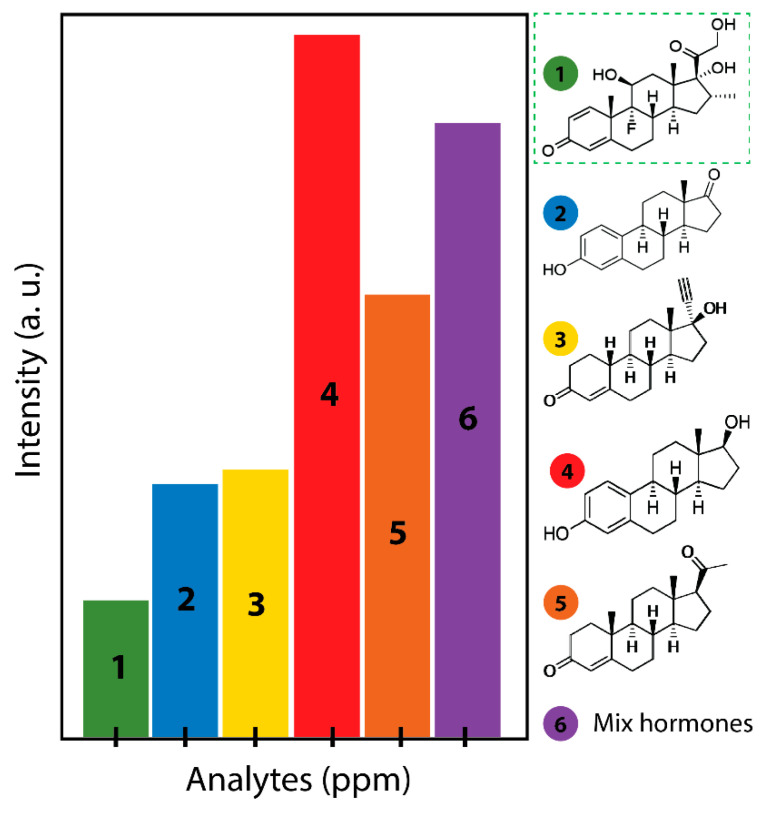
Specificity and selectivity for dexamethasone: different hormone analogs of dexamethasone interaction with rGO/PEI/Flu-DEX-apt under the same experimental conditions (1- dexamethasone; 2-estrone; 3- 19-norethindrone; 4- estradiol; 5-progesterone; 6- mixed hormones); the fluorescence peaks measured from different hormones at 15 ppm.

To demonstrate the selectivity of the prepared membrane to dexamethasone, analog hormones with a similar chemical structure were tested. The cross-reactivity was tested by using progesterone, estrone, estradiol, 19-norethindrone, and a mixture of hormones. An extremely low intensity of the dexamethasone fluorescence was observed compared with the progesterone, estrone, estradiol, 19-norethindrone, and mixture of hormones (Figure 5). Such data (Figure 4 and Figure 5) indicated that the hormone-like dexamethasone binds with high specificity and selectivity to the fabricated membrane. Several studies have demonstrated the absorption capacity of rGO foam/ polymer (PEI / polyethylene glycol) for pharmaceutical contaminants such as 17β-estradiol [40] or toxic cations from water [41]. However, these studies are not based on the importance of the affinity and specificity of the adsorption of contaminants by using a biomolecule such as aptamers. Our innovative approach made it possible to target the steroid molecules of interest that could adsorb with high affinity and above all specificity to a 3D membrane.

## 4. Conclusions

We designed a specific membrane by using reduced graphene oxide, polyethyleneimine, dexamethasone-aptamer as proof of the concept for dexamethasone hormone removal. The affinity and the specificity of aptamers to decontaminate water-based 3D graphitic foam (membrane) were tested. The fact that graphene oxide is well known as a fluorescence quencher was used as a key to follow and to confirm the adsorption steps for the analyte in the membrane. We found that the efficiency of the aptamer–analyte binding drastically decreased the fluorescence intensity, the association between the 3D membrane and the ssDNA (aptamer) was directly affected by the varying graphene oxide concentrations. The GO concentrations affected the pores’ size formation. Our results showed that the highest concentration of graphene oxide solution yielded ordered porosity with large diameters. The chelation and removal of the dexamethasone-ssDNA were confirmed and tested for several hormonal analogs. The resulting rGO 3D membrane functionalized with aptamer could be valuable for designing and optimizing many specific membranes for different applications in various disciplines in water treatment, such as sewage purification and desalination, due to its high mechanical strength, superior flexibility and hydrophilic property.
